# The two sides of opioids: The subtle boundary between medication and abuse

**DOI:** 10.1016/j.eclinm.2022.101538

**Published:** 2022-06-22

**Authors:** eClinicalMedicine

Opioid overuse is causing concerns, which until recently were thought to be restricted to the USA, where deaths due to opioid misuse have quadrupled due to the abuse of prescribed opioids. Indeed, the World Drug Report from 2021 has reported that the prevalence of opioid use from 2010 to 2019 has increased in North Africa, Central Asia, Transcaucasia (Armenia and Georgia), Middle East, South-West Asia, and South Asia, and Western and Central Europe, while it has slightly decreased in North and South America, Eastern and South-Eastern Asia, Eastern and South-Eastern Europe, and Oceania.

What exactly are opioids and how are they changing the lives of so many people? Opioids are a group of natural compounds like morphine and codeine extracted from opium poppy seeds. They can be also chemically synthesised to make heroin, methadone, and tramadol. Opioids bind to the opioid receptors mainly expressed in the brain, spinal cord, peripheral neurons, and gut. Within these target sites they exert an analgesic effect. Opioids are commonly prescribed medications used to treat chronic pain—a global health problem affecting 30% of people worldwide. However, opioids do not only have a beneficial effect, indeed, like many other drugs, they can cause several side effects, including sedation, dizziness, and sleepiness.

The main problem related to opioid use relies on their prolonged administration, which in most cases, can lead to addiction in the patient. When opioids bind to their receptors, the release of endorphins causes a temporary pleasant feeling, once gone evokes an overwhelming desire to take more drugs. In addition, prolonged opioid administration can cause tolerance to the drug, resulting in a constant need to increase their dosage. The misuse of opioids is reported to causes 0·35 million deaths per year—attributed to drug use disorders with overdose in 30% of the cases worldwide.

In the last 20 years, opioid overdose deaths in North America have risen due to the so-called opioid crisis, making the start of the largest opioids epidemic that continues to grow worldwide. In the USA, opioids have not only been prescribed to patients affected by severe pain, but they have also frequently been prescribed to patients suffering with headache or backpain; consequently the total number of people exposed to opioids has soared, as have addiction and dependence. Furthermore, this overprescription trend coincides with a rise of overdose deaths, mainly caused by fentanyl and tramadol in the USA in 2010–19. The same trend has been observed in England, where opioid prescription increased by 34% between 1998 and 2016. It has been estimated that the global numbers of people who use opioids from 2010 to 2019 increased from 30 million to 60 million.

Opioids are produced by only three countries, Afghanistan (83% of the global production), Myanmar (7%), and Mexico (6%). In the period of 1998–2020, the trade of opioids has grown up to double and continues expanding in response to the high use of opioids. Moreover, because opioids can be easily obtained by prescription or by online purchase, there has been a switch of the opioid purchase from the usage of illegal opioids, like heroin, to pharmaceutical opioids, such as oxycodone, morphine, codeine, and fentanyl. Similar switch has occurred for example in the United Arab Emirates where tramadol is now the most common opioid used as medication. Additionally in Sweden, Estonia, and Finland, opioid misuse is increasing—highlighting the severe addictive side effect of opioids and prescription drugs of such.

Opioid misuse remains a crucial problem facing many countries worldwide. The world must fight it by drastically restricting opioids use and by reducing the number of people who are addicted. Still a lot of work must be done to tackle the rising death toll caused by opioids overdose. Governments and physicians are called to action to stop the overprescription and with that the entry mechanism into a long-term addiction for patients. New strategies need to be implemented to limit the misuse of opioids for minor pain management and to restrict the medical prescription of opioids to severe conditions alone.

